# Long-term outcomes of biological mesh repair following extra levator abdominoperineal excision of the rectum: an observational study of 100 patients

**DOI:** 10.1007/s10151-019-02056-0

**Published:** 2019-08-07

**Authors:** P. W. Thomas, J. E. M. Blackwell, P. J. J. Herrod, O. Peacock, R. Singh, J. P. Williams, N. G. Hurst, W. J. Speake, A. Bhalla, J. N. Lund

**Affiliations:** 1 Department of Colorectal Surgery, Royal Derby Hospital, Derby, DE22 3NE UK; 2 Department of Radiology, Royal Derby Hospital, Derby, DE22 3NE UK; 3 Medical Research Council-Arthritis Research UK Centre for Musculoskeletal Ageing Research, University of Nottingham, Royal Derby Hospital, Derby, DE22 3DT UK

**Keywords:** Abdominoperineal excision, Surgical mesh, Rectal cancer, Hernia

## Abstract

**Background:**

Current evidence suggests that pelvic floor reconstruction following extralevator abdominoperineal excision of rectum (ELAPER) may reduce the risk of perineal herniation of intra-abdominal contents. Options for reconstruction include mesh and myocutaneous flaps, for which long-term follow-up data is lacking. The aim of this study was to evaluate the long-term outcomes of biological mesh (Surgisis^®^, Biodesign™) reconstruction following ELAPER.

**Methods:**

A retrospective review of all patients having ELAPER in a single institution between 2008 and 2018 was perfomed. Clinic letters were scrutinised for wound complications and all available cross sectional imaging was reviewed to identify evidence of perineal herniation (defined as presence of intra-abdominal content below a line between the coccyx and the lower margin of the pubic symphysis on sagittal view).

**Results:**

One hundred patients were identified (median age 66, IQR 59–72 years, 70% male). Median length of follow-up was 4.9 years (IQR 2.3–6.7 years). One, 2- and 5-year mortality rates were 3, 8 and 12%, respectively. Thirty three perineal wounds had not healed by 1 month, but no mesh was infected and no mesh needed to be removed. Only one patient developed a symptomatic perineal hernia requiring repair. On review of imaging a further 7 asymptomatic perineal hernias were detected. At 4 years the cumulative radiologically detected perineal hernia rate was 8%.

**Conclusions:**

This study demonstrates that pelvic floor reconstruction using biological mesh following ELAPER is both safe and effective as a long-term solution, with low major complication rates. Symptomatic perineal herniation is rare following mesh reconstruction, but may develop sub clinically and be detectable on cross-sectional imaging.

## Introduction

An abdominoperineal excision of the rectum (APER) may be required for patients with a rectal cancer less than 6 cm from the anal verge, where an anterior resection with anastomosis is not possible [1]. Concern over coning of the specimen with standard perineal dissection, leading to perforation and circumferential resection margin (CRM) involvement [2–6] with poorer oncological outcomes compared to a low anterior resection [7] led to the widespread adoption of extra-levator abdominoperineal excision of rectum (ELAPER).

The ELAPER technique, based upon the original description of APER by Miles [8], involves excision of a wide area of tissue around the rectal tumour, and division of the levators at their origin, producing a cylinder of tissue and avoiding wasting seen with standard technique [9]. This larger cylindrical specimen improves oncological outcome but leaves a large pelvic floor defect requiring reconstruction to avoid perineal herniation [10]. Wound complications are also increased by impaired healing associated with neoadjuvant radiotherapy to the low rectum used in the majority of these patients [11–13].

Several methods have been suggested for closure of the perineal defect, including primary closure (with [14] or without omentoplasty, [15]), myocutaneous flaps [11] and mesh repairs [16]. So far there is no clear consensus on the optimal method, with a lack of long-term follow-up data in this patient group [17]. Primary closure has been shown to increase wound tension and the potential for a postoperative collection due to the large amount of dead space left in the pelvis [13]. Myocutaneous flaps and biological mesh are currently recommended by specialty associations [17]. Flaps have been associated with higher costs, longer operating time, donor-site morbidity and the need to have an experienced plastic surgeon available [16]. We have previously shown that biological mesh reconstruction, first described by our group [18] is a safe and cost-effective method of perineal wound closure [16, 19].

Rates of perineal herniation (PH) vary widely in the current literature, occurring after primary closure, mesh and myocutaneous flap reconstruction [20–23]. It has been reported that the majority of perineal hernias occur in the first year postoperatively [21, 24, 25] but longer term follow-up is lacking.

The aim of this study was to evaluate long-term outcome data following a biological mesh pelvic floor reconstruction in a large cohort.

## Materials and methods

All patients having an ELAPER for low rectal cancer or a salvage procedure for anal cancer with biological mesh reconstruction were identified using a prospectively maintained colorectal multidisciplinary team database. This study adds additional cases and provides long-term follow-up to our earlier case series [19]. Any patient with disease extending beyond or involving the mesorectal fascia received neoadjuvant long course chemoradiotherapy (LCCRT) using 45 Gy in 25 fractions with twice daily capecitabine.

Intraoperative technique involved rectal dissection distally in the total mesorectal excision plane, until the mesorectum thins, before repositioning the patient into the prone jack-knife position and completing the extra-levator excision as previously described [16]. The perineal defect is then closed using biological mesh (Surgisis^®^, Biodesign™, Cook Medical, Bloomington, IN, USA), which is sutured laterally to the origins of the divided levators. There was no routine postoperative use of antibiotics or drains and the patients were allowed to mobilise immediately after surgery. Prolonged sitting was discouraged. Postoperatively, patients were followed up in a specialist-nurse led clinic 4 weeks after surgery, with 6-monthly appointments for 2 years and computed tomography (CT) scans of the chest, abdomen and pelvis at 1 and 2 years. A colonoscopy was also performed at 1 and 5 years. Some patients received longer follow-up due to individual circumstances related to either their perineal wound or progression of their disease.

Electronic case notes, clinic letters and cross-sectional imaging were reviewed to identify wound complications or symptoms of perineal herniation. Further review of cross-sectional imaging was performed to identify any evidence of perineal herniation. Perineal herniation was defined as the presence of intra-abdominal content beyond a line between the coccyx and the lower margin of the pubic symphysis on sagittal views.

Complications were defined as either early (≤ 30 days postoperatively) or late (> 30 days post-operatively) and graded according to the Clavien–Dindo classification [26]. A major complication was defined as Grade III or above of this classification system.

Descriptive data are presented as median (IQR) or *n* (%) as appropriate. No statistical analysis was performed for this descriptive study.

## Results

One hundred consecutive patients had biological mesh pelvic floor reconstruction after ELAPER between February 2008 and June 2018. The median age was 66.7 years (59–72). Seventy (70%) of the patients were male.

Demographics and preoperative staging are shown in Table 1, along with details of neoadjuvant treatment. The majority of patients (*n* = 70) had neoadjuvant long course chemoradiotherapy. One patient had a shortened course of chemotherapy due to side-effects.

**Table 1:** Patient demographics and preoperative data

Demographic	Number of patients *N* (%)
*Sex*	
Male	70 (70%)
Female	30 (30%)
Median age (range)	66.7 (39–83)
*ASA grade*	
1	32 (32%)
2	53 (53%)
3	14 (14%)
4	1 (1%)
*Preoperative staging*	
T-stage	
T1	4 (4.1%)
T2	27 (27.8%)
T3	53 (54.6%)
T4	13 (13.4%)
Nodal status	
N0	41 (42.3%)
N1	36 (37.1%)
N2	20 (20.6%)
Metastases	
M0	93 (95.9%)
M1	4 (4.1%)
Neoadjuvant therapy	
Long-course chemoradiotherapy	70 (70%)
Long-course radiotherapy	1 (1%)
None	29 (29%)
*Tumour location*	
Median distance from anal verge	2 cm
Range	0–6 cm

Histopathological findings are shown in Table 2, along with the details of any postoperative adjuvant chemotherapy received. Laparoscopic assistance during the abdominal phase of the procedure was used in 5 cases, with a further 4 cases using robotic assistance. The remaining cases were performed using the standard ELAPER technique as described previously [13]. There were 3 tumour perforations and 6 positive circumferential resection margins (CRM) in the 100 patients. Six patients had a local recurrence.

**Table 2:** Histopathological data

Histopathological data	Number of patients, *N* (%)
Adenocarcinoma	85 (85%)
SCC	4 (4%)
Melanoma	3 (3%)
*Staging*	
Complete response	12 (12%)
T1N0	11 (11%)
T1N1	1 (1%)
T2N0	24 (24%)
T2N1	6 (6%)
T2N2	2 (2%)
T3N0	25 (25%)
T3N1	10 (10%)
T3N2	6 (6%)
T4N0	1 (1%)
T4N1	1 (1%)
T4N2	1 (1%)
*Differentiation*	
Well	8 (8%)
Moderate	73 (73%)
Poor	4 (4%)
N/A	15 (15%)
*Lympho/vascular involvement*	
Yes	35 (35%)
No	65 (65%)
*CRM*	
Positive	6 (6%)
Negative	82 (82%)
N/A	12 (12%)
*Tumour perforation*	
Yes	3 (3%)
No	97 (97%)
*Post-operative therapy*	
Chemotherapy	29 (29%)
Chemoradiotherapy	4 (4%)
Lung resection	4 (4%)
Liver resection	5 (5%)

*SCC* squamous cell carcinoma

The median length of follow-up from time of operation was 4.9 (2.3–6.7) years with a median of 2.1 (1.3–2.8) years in which patients were seen in clinic. There was no death by 30 days and 1-, 2- and 5-year mortality was 3, 8 and 12%, respectively. Cumulative mortality was 22%, with only 1 patient dying from causes unrelated to rectal cancer.

### Perineal herniation

One patient developed a symptomatic perineal hernia, presenting with a painful palpable perineal lump that required elective surgical repair. This hernia was confirmed on CT at 379 days following ELAPER.

This perineal hernia was repaired with the patient in the jack-knife prone position. The perineal scar was reopened and the sac carefully dissected down to the pelvic sidewall at the level of the levator attachments. The sac was bivalved and one leaf edge sutured to the opposite pelvic side-wall. A new mesh was fixed to the pelvic sidewall laterally and posteriorly, and the remaining sac secured to the opposite side wall, sandwiching the mesh between the two.

**Fig. 1:**
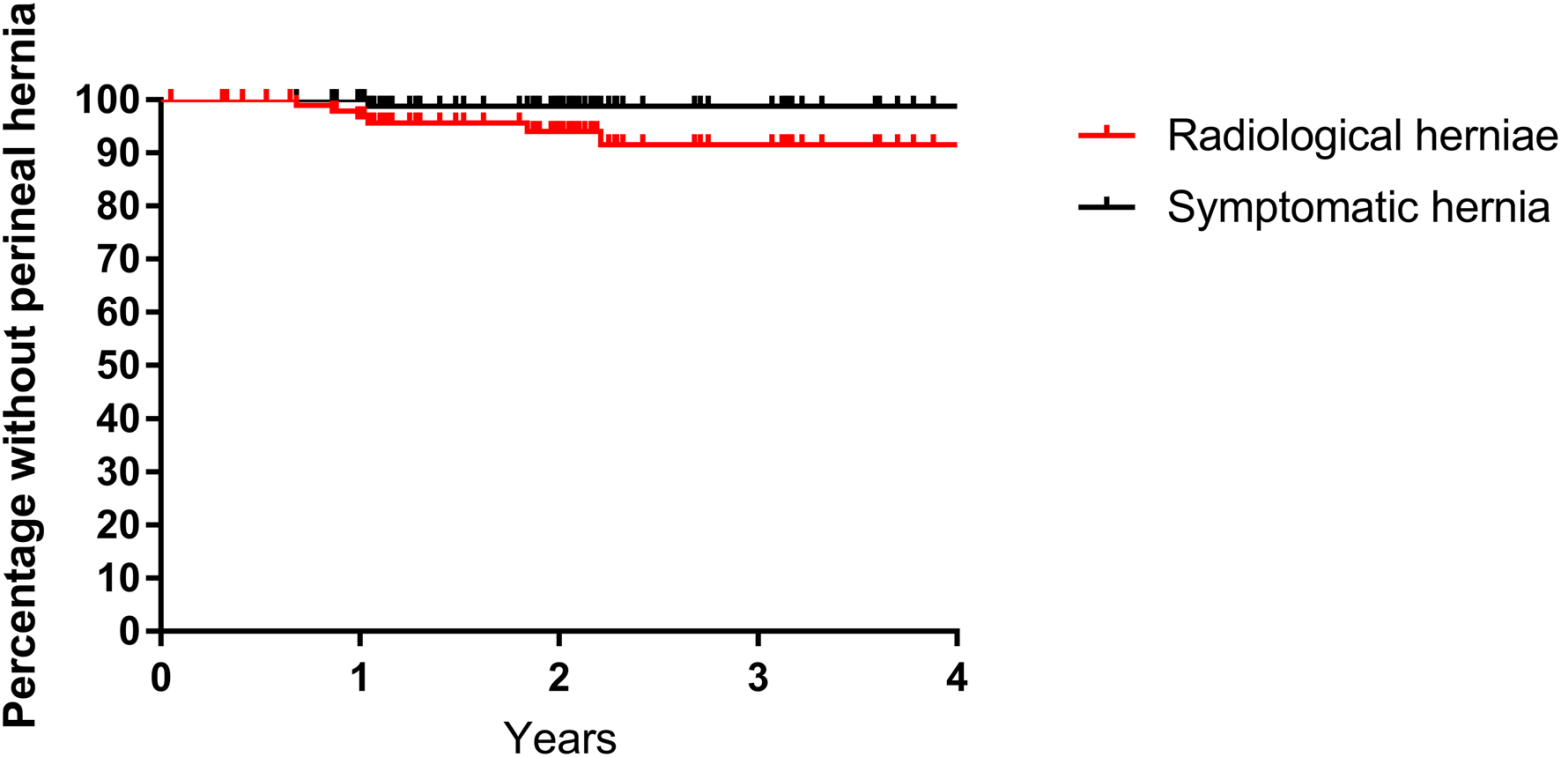
Kaplan–Meier curve of radiologically detected and symptomatic perineal herniae

A further 7 asymptomatic perineal hernias, not detectable on clinical examination, were discovered radiologically on retrospective review of CT images. Only 1 of these 7 hernias was mentioned in the original CT report. Figure 1 displays a Kaplan–Meier curve of both radiologically detected and symptomatic perineal herniae. At 4 years the cumulative radiologically detected perineal hernia rate was 8%.

### Other complications

No other complications were directly attributable to the biological mesh and no mesh needed to be removed. Table 3 describes the wound complications, which occurred in 33/100 patients. There were only 7 (7%) major wound complications.

**Table 3:** Wound Complications

Wound complication	Number of patients, *N* (%)	Clavien–Dindo classification
Delayed wound healing	33 (33%)	I
Simple discharge requiring dressing	8 (8%)	I
Sinus formation	5 (5%)	I
Partial dehiscence	4 (4%)	I
Superficial wound infection requiring antibiotics	9 (9%)	II
Dehiscence requiring EUA	3 (3%)	IIIb
Collection/abscess requiring VAC	4 (4%)	IIIb

*EUA* examination under anaesthesia, *VAC* vacuum assisted closure

The most common complication unrelated to the perineal wound was parastomal hernia, which was seen in 17/100 patients (Table 4). One of these parastomal hernias required repair. Eight patients continue to suffer from chronic perineal pain; which has been extensively investigated in each with no cause identified. There was no postoperative death as a result of complications.

**Table 4:** Other complications

Type of complication	Number of patients	Clavien–Dindo classification
*Early*		
Hospital acquired pneumonia	2 (2%)	II
Ileus	2 (2%) (1 required TPN)	II
Small bowel obstruction	3 (3%)	II
Pulmonary embolism	1 (1%)	II
*Late*		
Parastomal hernia	17 (17%)	I
Chronic perineal pain	8 (8%)	I
Chronic back pain	1 (1%)	I
Incisional hernia	2 (2%)	I
Perineal hernia	7 (7%)	I
Deep vein thrombosis	1 (1%)	II
Urinary incontinence	1 (1%)	II
Parastomal hernia requiring repair	1 (1%)	IIIb
Perineal hernia requiring repair	1 (1%)	IIIb

*TPN* total parenteral nutrition

## Discussion

This is the largest series of biological mesh perineal reconstruction following ELAPER. This study shows biological mesh repair is an effective and safe method for the perineal reconstruction following ELAPER, with no mesh related early complications and a very low long-term herniation rate.

One of the main challenges to closing the perineal wound after ELAPER is the impairment of healing as a consequence of preoperative long course chemoradiotherapy, which doubles the rate of perineal wound complications in ELAPER [12, 27], probably due to impaired tissue oxygenation, decreased fibroblast production of collagen and altered cellular response [13]. Perineal wound infection rates have been reported to be as high as 37% following mesh repair [28]. Our study showed 33% of patients had a wound that was not completely healed at 1 month, consistent with the current literature, which reports delayed healing rates of up to 42% [23, 29–32]. Due to the position of insertion; the biological mesh rarely becomes involved in a superficial wound infection, and as such removal of the mesh is highly unlikely to be required [20, 28]. Seven patients had a wound dehiscence that required examination under anaesthesia (EUA), with 4 of these needing vacuum-assisted closure (VAC).

Wide variation in perineal hernia rates is reported in the literature. A recent systematic review reports rates of 0–26%, with variable lengths of follow-up [24]. In addition, there is currently no standard definition of a perineal hernia following ELAPER. There may be underreporting of asymptomatic perineal hernias on CT, as demonstrated in this study, because the scan is performed as part of surveillance rather than specifically focusing on the level of the pelvic floor [25]. Of the 8 hernias that were identified, just 2 were mentioned in the standard report and the remaining 6 identified only retrospectively as part of this study. Whether radiologically detected hernias progress to symptomatic hernias requiring treatment is unknown.

Most perineal hernias are thought to occur within the first 1–2 years after surgery, and this is supported by our findings [28, 31]. The biological mesh used is an acellular matrix biomaterial produced from porcine small intestine mucosa. It encourages the proliferation and the formation of fibroblasts in the wound, without triggering the body’s natural response to a foreign body [33]. Once inserted it is quickly incorporated, resulting in strong vascularised tissues [34]. Although Surgisis^®^ is not a cross-linked mesh [35], raising concerns regarding the use of this mesh in high-tension areas [36], the current study has shown it provides adequate support in 92% of patients.

Other methods have been proposed aiming to reduce the incidence of perineal hernia. Recently, Blok et al. described the use of omentoplasty [15]. However, this showed no decrease in the pelvic or perineal morbidity and increased the risk of reoperation in those having omentoplasty. Bulut et al. proposed a technique of using a large filled catheter balloon in the pelvic cavity with the intention of forming a thin fibrotic peritoneal layer [37]. No perineal hernias were detected by 3 years in a small cohort of 15 patients [37].

The most frequently used alternatives to mesh repair are myocutaneous flaps [17]. Flaps provide support in a high tension area, also with the aim of importing non-irradiated tissue to aid healing [13]. A systematic review did not identify any difference in perineal wound complications and hernias between mesh and myocutaneous flaps [17, 38]. However, Christensen et al. did find a large difference in perineal hernia rates between fasciocutaneous flaps (21%) and mesh reconstruction (0%) [21]. There are significant financial costs associated with myocutaneous flap compared to biological mesh reconstruction that need to be considered, primarily due to the increased operative costs and length of hospital stay [16]. Patients are also not required to be on their side lying down for 6 weeks if a mesh reconstruction is performed.

A wide variation in wound healing and perineal hernia rates is also reported after primary closure of the perineum [17]. Primary closure is associated with high tension and a large volume area of dead space, which predisposes to postoperative perineal complications [39]. The only RCT comparing primary closure with biological mesh reconstruction, showed no significant difference in wound healing, but a large difference in perineal hernia rates. The clinically detectable perineal hernia rate was 27% in the primary closure group compared with 13% in the biological mesh group (*p* = 0.0316) [14]. Sayers et al. reported 5-year outcomes following ELAPER and showed a 26% perineal hernia rate [25]. In primary closure, they also reported a mean time to perineal hernia of 10.5 months [25].

## Conclusions

Biological mesh is both a safe and effective long-term method for perineal reconstruction after extralevator abdominoperineal excision of the rectum, and is associated with a very low rate of perineal herniation.

## Data Availability

The datasets analysed during the current study are available from the corresponding author on reasonable request.

## References

[1] Wibe A, Syse A, Andersen E (2004). Oncological outcomes after total mesorectal excision for cure for cancer of the lower rectum: anterior vs. abdominoperineal resection. Dis Colon Rectum.

[2] West NP, Finan PJ, Anderin C (2008). Evidence of the oncologic superiority of cylindrical abdominoperineal excision for low rectal cancer. J Clin Oncol.

[3] West NP, Anderin C, Smith KJE (2010). Multicentre experience with extralevator abdominoperineal excision for low rectal cancer. Br J Surg.

[4] Eriksen MT, Wibe A, Syse A (2004). Inadvertent perforation during rectal cancer resection in Norway. Br J Surg.

[5] Negoi I, Hostiuc S, Paun S (2016). Extralevator vs conventional abdominoperineal resection for rectal cancer—a systematic review and meta-analysis. Am J Surg.

[6] Bianco F, Romano G, Tsarkov P (2017). Extralevator with *vs* nonextralevator abdominoperineal excision for rectal cancer: the RELAPe randomized controlled trial. Color Dis.

[7] Mauvais F, Sabbagh C, Brehant O (2011). The current abdominoperineal resection: oncological problems and surgical modifications for low rectal cancer. J Visc Surg.

[8] Miles WE (1971). A method of performing abdomino-perineal excision for carcinoma of the rectum and of the terminal portion of the pelvic colon (1908). CA Cancer J Clin.

[9] Holm T, Ljung A, Häggmark T (2007). Extended abdominoperineal resection with gluteus maximus flap reconstruction of the pelvic floor for rectal cancer. Br J Surg.

[10] Bignell M, Chave H, Branagan G (2018). Outcome of surgery for recurrent anal cancer: results from a tertiary referral centre. Color Dis.

[11] Howell AM, Jarral OA, Faiz O (2013). How should perineal wounds be closed following abdominoperineal resection in patients post radiotherapy—primary closure or flap repair? Best evidence topic (BET). Int J Surg.

[12] Bullard KM, Trudel JL, Baxter NN, Rothenberger DA (2005). Primary perineal wound closure after preoperative radiotherapy and abdominoperineal resection has a high incidence of wound failure. Dis Colon Rectum.

[13] Nisar PJ, Scott HJ (2009). Myocutaneous flap reconstruction of the pelvis after abdominoperineal excision. Color Dis.

[14] Musters GD, Klaver CEL, Bosker RJI (2017). Biological mesh closure of the pelvic floor after extralevator abdominoperineal resection for rectal cancer. Ann Surg.

[15] Blok RD, de Jonge J, de Koning MA (2019). Propensity score adjusted comparison of pelviperineal morbidity with and without omentoplasty following abdominoperineal resection for primary rectal cancer. Dis Colon Rectum.

[16] Peacock O, Pandya H, Sharp T (2012). Biological mesh reconstruction of perineal wounds following enhanced abdominoperineal excision of rectum (APER). Int J Colorectal Dis.

[17] Foster JD, Tou S, Curtis NJ (2018). Closure of the perineal defect after abdominoperineal excision for rectal adenocarcinoma—ACPGBI Position Statement. Color Dis.

[18] Boereboom CL, Watson NFS, Sivakumar R (2009). Biological tissue graft for pelvic floor reconstruction after cylindrical abdominoperineal excision of the rectum and anal canal. Tech Coloproctol.

[19] Peacock O, Simpson JA, Tou SI (2014). Outcomes after biological mesh reconstruction of the pelvic floor following extra-levator abdominoperineal excision of rectum (APER). Tech Coloproctol.

[20] Dinnewitzer A, Meissnitzer M, Meissnitzer T (2015). Dynamic magnetic resonance imaging evaluation of pelvic reconstruction with porcine dermal collagen mesh following extra-levator abdominoperineal excision for primary rectal cancer. Int J Colorectal Dis.

[21] Henrik Kidmose Christensen PNTTSL (2011). Perineal repair after extralevator abdominoperineal excision for low rectal cancer. Dis Colon.

[22] Butt HZ, Salem MK, Vijaynagar B (2013). Perineal reconstruction after extra-levator abdominoperineal excision (eLAPE): a systematic review. Int J Colorectal Dis.

[23] Harries RL, Luhmann A, Harris DA (2014). Prone extralevator abdominoperineal excision of the rectum with porcine collagen perineal reconstruction (Permacol^TM^): high primary perineal wound healing rates. Int J Colorectal Dis.

[24] Balla A, Batista Rodríguez G, Buonomo N (2017). Perineal hernia repair after abdominoperineal excision or extralevator abdominoperineal excision: a systematic review of the literature. Tech Coloproctol.

[25] Sayers AE, Patel RK, Hunter IA (2015). Perineal hernia formation following extralevator abdominoperineal excision. Color Dis.

[26] Dindo D, Demartines N, Clavien P-A (2004). Classification of surgical complications: a new proposal with evaluation in a cohort of 6336 patients and results of a survey. Ann Surg.

[27] Musters GD, Buskens CJ, Bemelman WA, Tanis PJ (2014). Perineal wound healing after abdominoperineal resection for rectal cancer: a systematic review and meta-analysis. Dis Colon Rectum.

[28] Schiltz B, Buchs NC, Penna M (2017). Biological mesh reconstruction of the pelvic floor following abdominoperineal excision for cancer: a review. World J Clin Oncol.

[29] Jones H, Moran B, Crane S (2017). The LOREC APE registry: operative technique, oncological outcome and perineal wound healing after abdominoperineal excision. Color Dis.

[30] Aslam MI, Baloch N, Mann C (2019). Simultaneous stoma reinforcement and perineal reconstruction with biological mesh—a multicentre prospective observational study. Ann Med Surg.

[31] Musters GD, Bemelman WA, Bosker RJ (2014). Randomized controlled multicentre study comparing biological mesh closure of the pelvic floor with primary perineal wound closure after extralevator abdominoperineal resection for rectal cancer (BIOPEX-study). BMC Surg.

[32] Anderin C, Martling A, Lagergren J (2012). Short-term outcome after gluteus maximus myocutaneous flap reconstruction of the pelvic floor following extra-levator abdominoperineal excision of the rectum. Color Dis.

[33] Konstantinovic ML, Lagae P, Zheng F (2005). Comparison of host response to polypropylene and non-cross-linked porcine small intestine serosal-derived collagen implants in a rat model. BJOG.

[34] Ayubi FS, Armstrong PJ, Mattia MS, Parker DM (2008). Abdominal wall hernia repair: a comparison of Permacol^®^ and Surgisis^®^ grafts in a rat hernia model. Hernia.

[35] Sainfort A, Denis Hallouard I, Hartmann D (2016). Xenograft biologic mesh in parietal and general surgery: technical assessment and review of clinical effectiveness and safety data. J Visc Surg.

[36] Pascual G, Sotomayor S, Rodríguez M (2015). Extraperitoneal and intraperitoneal behavior of several biological meshes currently used to repair abdominal wall defects. J Biomed Mater Res Part B Appl Biomater.

[37] Bulut O, Rasmussen HB, Jess P (2012). Use of a balloon catheter in management of the pelvic space following laparoscopic abdominoperineal excision. Color Dis.

[38] Foster JD, Pathak S, Smart NJ (2012). Reconstruction of the perineum following extralevator abdominoperineal excision for carcinoma of the lower rectum: a systematic review. Color Dis.

[39] Lee P, Tan WJ, Brown K, Solomon MJ (2018). Addressing the empty pelvic syndrome following total pelvic exenteration: does mesh reconstruction help?. Color Dis.

